# Distinct relationships between social aptitude and dimensions of manic-like symptoms in youth

**DOI:** 10.1007/s00787-015-0800-7

**Published:** 2015-12-09

**Authors:** Xavier Benarous, Nina Mikita, Robert Goodman, Argyris Stringaris

**Affiliations:** 1 King’s College London, Institute of Psychiatry, Psychology and Neuroscience, Box P085, De Crespigny Park, Denmark Hill, London, SE5 8AF UK; 2 Department of Child and Adolescent Psychiatry, Hôpital Pitié-Salpêtrière, 47-83, Boulevard de l’Hôpital, 75013 Paris, France; 3 19 rue de Turenne, 75004 Paris, France

**Keywords:** Manic symptoms, Paediatric bipolar disorder, Factor analysis, Social aptitude, Social skills

## Abstract

Difficulties with interpersonal relationships have been reported in children and adolescents with manic symptoms, even if they do not fulfil criteria for a manic episode. The role of social aptitude (SA) in youths with manic symptoms has never been examined in the general population. Moreover, no study has examined whether SA is differentially associated with dimensions of manic symptoms. We hypothesised that youth with predominantly undercontrol manic symptoms (characterised by irritability) would show lower levels of SA; conversely, youth with predominantly exuberant symptoms would show better than average social skills. Our sample comprised 5325 participants from the 2004 British Child and Adolescent Mental Health Survey (B-CAMHS04), mean age 10.3 years, SD = 3.3, 48 % girls. Manic symptoms were assessed with the Development and Wellbeing Assessment by interviewing parents and young people. Children and adolescents with manic symptoms had a lower SA score, compared to the general population by parent report, but not by self-report. SA score was higher in youths with predominantly exuberant manic symptoms compared to the general population; whereas the youths with predominantly undercontrol manic symptoms had lower SA scores by parent and self-report. Our results provide further evidence for the distinction between exuberant and undercontrol manic symptoms and highlight the need to focus on SA in future research.

**Electronic supplementary material:**

The online version of this article (doi:10.1007/s00787-015-0800-7) contains supplementary material, which is available to authorized users.

## Introduction

A number of studies show that mania is associated with substantial social impairment in adults [1] and in children [2–6]. The origins of this impairment are not entirely clear but may stem from a variety of sources. First, negatively valenced affect, such as irritability during a manic episode could alienate peers and lead to social difficulties [7–9]; second, the intensity of emotional arousal may decrease levels of social competence [10, 11]; third, underlying deficits in executive functioning in those with bipolar disorder may also contribute to diminished social competence [12–18]. However, some recent studies also suggest that certain patients with bipolar disorder may perform better on social cognitive tasks than non-affected controls [19–21] and report higher levels of sociability in bipolar co-twins compared to controls [22].

The question, therefore, arises about what could underlie these apparently contradicting findings in the relationship between mania and social competence. We propose that these could be explained by heterogeneity within manic symptoms.

Using factor analysis of the self-administered hypomania checklist-32 (HCL-32), two distinct dimensions were identified in adults [23, 24]. The active-elated dimension was associated with fewer comorbid psychiatric disorders and less perceived stress; while an “irritable/risk-taking” dimension was linked with a low score on measures of well-being and more psychopathology. A comparable two-factor structure was found in both children and adolescents [25–28]. Holtmann et al. distinguished between “active-elated” symptoms that were associated with good psychosocial adjustment, in contrast to dimensions of “irritable-erratic” and “disinhibited” symptoms that were related to disruptive behaviour disorders in adolescents [25]. Topor also reported two dimensions of manic symptoms, namely an “activated/pleasure seeking” dimension and a “labile/disorganized” dimension (in children) and a “disorganized/psychotic” (in adolescents) [26]. The two-factor structure was replicated in a large community sample of 2512 Brazilian children from 6 to 12 years old [28]. In a study based on the 2004 British Child and Adolescent Mental Health Survey (B-CAMHS04), it was possible to distinguish an “exuberant” dimension linked to high activity and hedonistic behaviours (e.g. joking more than usual, more outgoing than usual, feeling overconfident) and an “undercontrol” dimension related to a low level of emotion regulation (e.g. being more irritable, more likely to take a serious risk) [27]. The undercontrol, but not the exuberant dimension, was associated with a low level of general functioning and higher rates of psychopathology (emotional disorder, disruptive disorder, attention-deficit disorder) [25–28]. The negative association between undercontrol dimension and the level of functioning remained significant after adjusting for the presence of DSM-IV disorders or dimensional measures of psychopathology. In another sample, the undercontrol, but not the exuberant, dimension was associated with poor performance on a response inhibition task [29]. By contrast, the exuberant dimension was found to be associated with a higher verbal IQ than in healthy children [29].

Based on these findings our study sets out to test the assumption that heterogeneity in manic symptoms could be useful in reconciling apparently contradicting findings about social aptitude in youth with bipolar disorder. For this purpose we use an epidemiological sample that allows us to study manic symptoms as a dimension across the general population of young people and is free of referral and Berkson biases. Our study focuses on social aptitude, which encompasses the personal abilities underlying social competence, especially the capacity to encode and interpret social cues to draw inferences about other people’s beliefs and intentions [30]. This has the advantage of tapping a more uniform construct than “social competence” which has been criticised for being overinclusive and containing aspects such as situational social or societal characteristics [8, 31–33].

We test the following hypotheses:

In keeping with previous studies we expect to find overall reduced social aptitude to be associated with manic symptoms in young people.

However, we hypothesise that social aptitude will be differentially associated with the exuberant and the undercontrol symptom dimensions. In particular, we expect that the undercontrol dimension will be associated with reduced social aptitude in youth. This expectation stems from the clinical and cognitive characteristics of this dimension. Children with symptoms of undercontrol are characterised by irritable and reckless behaviours which may negatively impact on social aptitude [25–28]. Moreover, they are characterised by increased cognitive impulsivity which could be a further hindrance to social interactions [29, 34]. By contrast, we expect that the exuberant dimension will be associated with higher-than-average social aptitude in youth. This is based on findings about the facilitative effects that positive mood valence may have on social interactions [7]. In addition, the higher-than-average verbal abilities of youth with symptoms of exuberance may promote social interactions [29, 35].

## Materials and methods

### Population

The 2004 British Child and Adolescent Mental Health Survey (B-CAMHS04) comprised a sample of 5–16 year olds (*n* = 7977) representative of the general British population; and has previously been described in detail [36]. The study used “child benefit” (a universal state benefit that at the time was payable in Great Britain for each child in a family) to develop a sampling frame of 5–16-year-olds in different postal sectors in England, Wales, and Scotland. After excluding families with no recorded postal code, it was estimated that this represented 90 % of all British children. Out of the 12,294 contacted, there were *n* = 1085 who opted out and* n* = 713 who were not eligible or had moved without trace, leaving 10,496 who were approached in person. Of those, *n* = 7977 participated (65 % of those selected; 76 % of those approached), and *n* = 5325 (67 %) participated in the second wave in 2007. As the bipolar module was only added in the 2007 survey, data reported are all from 2007. Parents and children above 7 years were presented with the following preamble: “Some young people have episodes of going abnormally high. During these episodes they can be unusually cheerful, full of energy, speeded up, talking fast, doing a lot, joking around, and needing less sleep. These episodes stand out because the young person is different from their normal self.” They were then asked: “Do you [Does X] ever go abnormally high?” to which they had the options of answering: “no”, “a little”, “a lot”. By parent report, 627 replied “a little” or “a lot” to the screening question. By youth report, 913 replied “a little” or “a lot” to the screening question. Those who answered “a little” or “a lot” to the screening question have to complete the DAWBA bipolar module. For this screening question, those answering “A little” had significantly more comorbidity and social impairment than those answering “No” [27]. In the interest of having as broad as possible a representation of subjects, we chose to include as “screen positive” those who answered “A little” as well as those who answered “A lot”. The DAWBA bipolar module was completed by 93 % (583/627) of participating parents and 96 % (874/913) of participating youths. There were no statistically significant differences in rates of overall psychopathology between those who completed the module and those who did not (*χ*^2^ = 0.42, *p* = 0.52, by parent report; *χ*^2^ = 3.31, *p* = 0.07, by self-report). Given the weak evidence that incomplete responders differed from complete responders, we used listwise deletion to deal with missing data. The main clinical characteristics of this sample have previously been described [27]. Throughout this paper, the findings for parent report and youth report on symptoms are presented separately.

### Measures

The Development and Well-Being Assessment (DAWBA) was used in the survey [37, 38]. It is a structured interview administered by lay interviewers with questions that are closely related to the diagnostic criteria of the DSM-IV. It focuses on current rather than life-time problems and was used to make DSM-IV-based diagnoses. The variable “any psychiatric disorders” reflects the presence of at least one disorder among: anxiety disorders depressive disorders, disruptive disorders, attention-deficit disorders, autistic spectrum disorders, eating disorders and tics disorders. The κ-statistic for chance-corrected agreement between two raters was 0.86 for any disorder (SE 0.04), 0.57 for internalizing disorders (SE 0.11) and 0.98 for externalizing disorders (SE 0.02) [37]. The DAWBA interview was administered to all parents and to all youth aged 11 or more.

The DAWBA Bipolar Module inquired as to whether the youth had ever had an episode “of going abnormally high.” Those who endorsed a previous abnormally high mood episode were then asked whether any of 26 additional manic symptoms [39] also occurred during episodes when they were feeling “abnormally high” (Table S1). For each individual symptom, the participants had the option of choosing between one of the following answers: “no”, “a little”, “a lot”. Given that the two first answers did not differ with respect to their prediction of overall impairment, we dichotomised each item by merging the “no” and “a little” responses into a “no” group (versus “a lot”). Re-running analyses using the trichotomous item coding did not alter the key findings of this paper. Information concerning the frequencies of individual items, the predictive validity and the factor structure of the DAWBA bipolar module has previously been presented [27]. The complete bipolar section of the interview can be seen at http://www.dawba.info/Bipolar/.

The Social Aptitudes Scale is a ten-item scale that forms part of the DAWBA (Table 1). It was designed to tap the sorts of social aptitude that require a good ability to read social and emotional cues rapidly in complex situations, to guide socially skilled behaviour [31]. Parents reported on how the child usually behaves in ten different social situations. The scale includes items such as “Able to compromise and be flexible”, “Easy to chat with, even if it isn’t on a topic that specially interests him/her” and “By reading between the lines of what people say, she/he can work out what they are really thinking and feeling”. For each item, parents rated their child as “a lot worse than average”, “a bit worse than average”, “about average”, “a bit better than average” or “a lot better than average”—the reference group being other children of the same age. The total sum of items ranges between 0 and 40. The scale has good reliability and validity [31]: internal consistency was found to be very good with Cronbach’s alpha of 0.88, and the total score was positively correlated with the prosocial subscale of the Strength and Difficulties Questionnaire (*r* = 0.42, *p* < 0.001). The distribution does not vary greatly by age. Copies of the Social Aptitudes Scale in many languages, along with the copyright terms for use, are available from http://dawba.info/SAS.

**Table 1:** The Social Aptitudes Scale

“How does [Name] compare with other children/people of his/her age in the following situations:”	
Item	Description
SAS1	Able to laugh around with others, for example accepting light-hearted teasing and responding appropriately
SAS2	Easy to chat with, even if it is not on a topic that specially interests him/her
SAS3	Able to compromise and be flexible
SAS4	Finds the right thing to say or do to defuse a tense or embarrassing situation
SAS5	Graceful when she/he does not win or get his/her own way. A good loser
SAS6	Other people feel at ease around him/her
SAS7	By reading between the lines of what people say, she/he can work out what they are really thinking and feeling
SAS8	After doing something wrong, she/he is able to say sorry and sort it out so that there are no hard feelings
SAS9	Can take the lead without others feeling they are being bossed about
SAS10	Aware of what is and is not appropriate in different social situations

The Social Aptitudes Scale is a copyright document belonging to Youthinmind Limited

The Strengths and Difficulties Questionnaire (SDQ) is a general measure of psychiatric symptomatology and impact in children. The SDQ impact score is a subscale generated for the parent- and self-report from the sum of 5 items: one item about distress; plus 4 items on social impairment in (a) family life, (b) friendships, (c) learning, and (d) leisure activities. The SDQ impact score demonstrated good internal consistency (Cronbach’s alpha of 0.88 for parent report, and 0.82 for self-report), with moderately strong correlation between informants.

### Statistical analyses

Our first question was whether social aptitude differs between children who screened positive for an episode of elated mood, as described above, compared to those who screened negative. Linear regressions were performed using the mean score on the Social Aptitudes Scale as a dependent variable, the presence of an episode of elated mood as an independent variable, with and without adjustment for age, gender, the presence of any DSM-IV psychiatric disorders, and the level of the SDQ impact score.

The second question of the paper was whether dimensions of mania symptoms were differentially related to social aptitude. The dimensional structure of the items of manic symptoms reported in the DAWBA bipolar module has previously been examined in children screened for an episode of elated mood [27]. A two-factor solution has been adopted based on indices of goodness-of-fit (i.e. Akaike information Criteria and Bayesian information Criteria). An exuberant dimension (Cronbach’s alpha = 0.84, eigen values = 12.52) and an undercontrol dimension (Cronbach’s alpha = 0.86, eigen values = 2.36) were identified. Weighted least squares means and variance adjusted were used to estimate the models followed by orthogonal rotation. The correlation between the two dimensions of manic symptoms was moderate (*r* = 0.59, *p* < 0.001). A similar structure was supported by studies conducted in two other samples [28, 29]. The second hypothesis was tested in a structural equation model with the social aptitude score as a dependent variable and the two latent dimensions of manic symptoms (undercontrol and exuberance) as predictors. All subjects who screened positively for an episode of elated mood and who completed the entire DAWBA bipolar module were included in the model (for parent report *n* = 583, for self-report *n* = 874). Distinct models were run for parent- and self-reported data. Re-running the analysis after excluding subjects with autistic spectrum disorder (*n* = 44) did not alter our findings. Structural equation models were performed using MPlus Version 5.

To interpret the results, we aimed to compare the social aptitude scores of youths with predominantly exuberant dimension of manic symptoms, to youths with predominantly undercontrol dimension of manic symptoms, to those presenting with both dimensions of symptoms, and finally to those who did not present with any manic symptoms (i.e. who screened negatively for an episode of elated mood). We generated sub-scales by summing the individual items designating the dimensions with factor loadings of 0.5 as episodic undercontrol and episodic exuberance. To identify groups of youths with relatively homogeneous dimensions of manic symptoms (i.e. predominantly undercontrol, or predominantly exuberant), we used the standardized difference between the sum score of undercontrol symptoms and the sum score of exuberant symptoms. Subjects labelled as “predominant undercontrol” presented a difference greater than or equal to 1; while subjects labelled as “predominant exuberant” presented a difference lesser than or equal to −1. Those with both undercontrol and exuberant types of symptoms (i.e. a difference score between −1 and +1) were labelled “Both types of symptoms”. A four-level categorical variable (no symptoms, predominant undercontrol, predominant exuberant, both types of symptoms) was created. To prevent differences between groups caused by an imbalance of demographic characteristics or comorbidity rates, we compared groups after matching the control group on age, gender, single-parent family status and the presence of any psychiatric disorders. We performed a one-way ANOVA to examine whether the mean social aptitude score was different between these four groups; and a Tukey’s HSD test to compare the mean social aptitude scores for each group. To facilitate the interpretations of results, we presented the standardized mean of the social aptitude score.

## Results

### Description of the sample screened positive for an episode of elated mood

Table 2 illustrates the main demographic and clinical characteristics of the sample. By parent report, 627 children (12 %) screened positive for an episode of elated mood. The mean age was 10.05 years, SD = 0.13, and 48 % were girls. Those screening positive for an episode of elated mood had a higher rate of psychopathology, and a higher SDQ impact score compared to those screened negative, Cohen’s *d* = 0.86. This was consistent with self-report findings (Cohen’s *d* = 0.86). Tables 3 and 4 present the demographic and clinical characteristics of each mania presentation groups (i.e. no manic-like symptoms, undercontrol predominant, exuberant predominant, and both types of symptoms) by parent and self-report.

**Table 2:** Clinical and sociodemographic characteristics of subjects screened positive and negative for elated episodes by parent and self-report

	Parent report			Self-report		
	Screening− *n* = 4630 88 %	Screening+ *n* = 627 12 %	Group differences	Screening− *n* = 2391 72 %	Screening-+ *n* = 913 28 %	Group differences
Socio-demographic characteristics						
Age, mean ± SD	10.36 **±** 0.05	10.05 **±** 0.13	*t* = 2.20, *p* = 0.028	11.67 **±** 0.050	11.60 **±** 0.076	*t* = 0.70, *p* = 0.486
Gender, female, *n* (%)	2237 (48 %)	304 (48 %)	*χ* ^2^ = 0.01, *p* = 0.936	1127 (47 %)	500 (55 %)	*χ* ^2^ = 15.39, *p* < 0.001
Ethnic group, white, *n* (%)	4193 (91 %)	558 (89 %)	*χ* ^2^ = 1.56, *p* = 0.212	2167 (91 %)	830 (91 %)	*χ* ^2^ = 0.06, *p* = 0.806
Parents not economically active, *n* (%)	492 (11 %)	113 (18 %)	*χ* ^2^ = 30.53, *p* < 0.001	241 (10 %)	95 (10 %)	*χ* ^2^ = 0.07, *p* = 0.792
Life events (more than 3), *n* (%)	397 (9 %)	117 (19 %)	*χ* ^2^ = 65.72, *p* < 0.001	230 (10 %)	121 (13 %)	*χ* ^2^ = 9.16, *p* = 0.003
DSM-IV disorders, *n* (%)						
Any disorder	300 (6 %)	173 (28 %)	*χ* ^2^ = 300.6, *p* < 0.001	152 (6 %)	114 (12 %)	*χ* ^2^ = 33.5, *p* < 0.001
Anxiety disorder	122 (3 %)	58 (9 %)	*χ* ^2^ = 73.0, *p* < 0.001	65 (3 %)	55 (6 %)	*χ* ^2^ = 20.6, *p* < 0.001
Depressive disorder	36 (1 %)	18 (3 %)	*χ* ^2^ = 23.8, *p* < 0.001	23 (1 %)	26 (3 %)	*χ* ^2^ = 16.0, *p* < 0.001
Attention-deficit disorder	32 (1 %)	38 (6 %)	*χ* ^2^ = 121.1, *p* < 0.001	18 (1 %)	13 (1 %)	*χ* ^2^ = 3.2, *p* = 0.073
Disruptive disorder	48 (1 %)	52 (8 %)	*χ* ^2^ = 218.4, *p* < 0.001	58 (2 %)	54 (6 %)	*χ* ^2^ = 24.5, *p* < 0.001
SDQ score, mean ± SD						
SDQ total score	6.81 ± 0.08	13.08 ± 0.29	*t* = −26.95, *p* < 0.001	8.78 ± 0.10	11.87 ± 0.17	*t* = −16.30, *p* < 0.001
Emotions symptoms	1.61 ± 0.03	3.07 ± 0.10	*t* = −17.77, *p* < 0.001	2.19 ± 0.04	3.04 ± 0.07	*t* = −11.75, *p* < 0.001
Conduct problem	1.24 ± 0.02	2.73 ± 0.09	*t* = −21.70, *p* < 0.001	1.73 ± 0.03	2.51 ± 0.06	*t* = −12.66, *p* < 0.001
Hyperactivity	2.74 ± 0.03	5.11 ± 0.11	*t* = −23.19, *p* < 0.001	3.52 ± 0.04	4.71 ± 0.07	*t* = −13.84, *p* < 0.001
Peer problem	1.22 ± 0.02	2.17 ± 0.08	*t* = −14.41, *p* < 0.001	1.33 ± 0.03	1.61 ± 0.05	*t* = −5.28, *p* < 0.001
Impact	0.28 ± 0.97	1.24 ± 2.03	*t* = −19.61, *p* < 0.001	0.14 ± 0.63	0.49 ± 1.12	*t* = −11.12, *p* < 0.001

**Table 3:** Clinical and sociodemographic characteristics of different mania presentation groups by parent report

Parent report	No manic-like symptoms *n* = 4630	Undercontrol predominant *n* = 86	Exuberant predominant *n* = 69	Both types of symptoms *n* = 412	Group differences
Socio-demographic characteristics					
Age, mean ± SD	10.38 ± 0.05	9.55 ± 0.33	10.61 ± 0.35	9.98 ± 0.16	*F* = *3.56*, *p* = *0.014*
Gender, female, *n* (%)	2269 (49 %)	33 (38 %)	37 (54 %)	206 (50 %)	*χ* ^2^ = *4.53*, *p* = *0.210*
Ethnic group, white, *n* (%)	4167 (90 %)	79 (92 %)	59 (86 %)	367 (89 %)	*χ* ^2^ = 2.09, *p* = 0.553
Parents not economically active, *n* (%)	509 (11 %)	17 (20 %)	7 (10 %)	78 (19 %)	*χ* ^2^ = 29.10, *p* < 0.001
Life events (more than 3), *n* (%)	417 (9 %)	29 (34 %)	12 (18 %)	66 (16 %)	*χ* ^2^ = 82.28, *p* < 0.001
DSM-IV disorders, *n* (%)					
Any disorder	278 (6 %)	52 (60 %)	5 (7 %)	87 (21 %)	*χ* ^2^ = 422.81, *p* < 0.001
Anxiety disorder	139 (3 %)	10 (12 %)	4 (6 %)	37 (9 %)	*χ* ^2^ = 63.34, *p* < 0.001
Depressive disorder	46 (1 %)	1 (1 %)	2 (3 %)	16 (4 %)	*χ* ^2^ = 29.26*, p* < 0.001
Attention-deficit disorder	46 (1 %)	24 (28 %)	0 (0 %)	400 (97 %)	*χ* ^2^ = 489.86, *p* < 0.001
Disruptive disorder	139 (3 %)	39 (45 %)	0 (0 %)	367 (89 %)	*χ* ^2^ = 428.57, *p* < 0.001
SDQ score, mean ± SD					
SDQ total score	6.81 ± 0.08	19.98 ± 0.71	8.29 ± 0.64	12.00 ± 0.31	*F* = 283.77, *p* < 0.001
Emotions symptoms	1.62 ± 0.03	4.15 ± 0.28	1.94 ± 0.25	2.88 ± 0.12	*F* = 100.18, *p* < 0.001
Conduct problem	1.25 ± 0.02	4.93 ± 0.26	1.58 ± 0.19	2.39 ± 0.10	*F* = 212.19, *p* < 0.001
Hyperactivity	2.74 ± 0.03	7.84 ± 0.22	3.06 ± 0.30	4.78 ± 0.13	*F* = 220.31, *p* < 0.001
Peer problem	1.20 ± 0.02	3.06 ± 0.23	1.71 ± 0.21	1.94 ± 0.09	*F* = 71.37, *p* < 0.001
Impact	0.27 ± 0.01	2.33 ± 0.23	0.59 ± 0.20	0.91 ± 0.08	*F* = 43.09, *p* < 0.001

**Table 4:** Clinical and sociodemographic characteristics of different mania presentation groups by self-report

Self-report	No manic-like symptoms *n* = 4405	Undercontrol predominant *n* = 134	Exuberant predominant *n* = 139	Both types of symptoms *n* = 600	Group differences
Socio-demographic characteristics					
Age, mean ± SD	10.07 ± 0.05	11.64 ± 0.19	11.57 ± 0.20	11.70 ± 0.09	*F* = 57.71, *p* = *0.0137*
Gender, female, *n* (%)	2070 (47 %)	66 (49 %)	79 (57 %)	336 (56 %)	*χ* ^2^ = 20.56*, p* = *0.210*
Ethnic group, white, *n* (%)	3964 (90 %)	126 (94 %)	117 (84 %)	546 (91 %)	*χ* ^2^ = 7.94, *p* = 0.0.47
Parents not economically active, *n* (%)	529 (12 %)	27 (20 %)	13 (9 %)	54 (9 %)	*χ* ^2^ = 16.60, *p* < 0.001
Life events (more than 3), *n* (%)	396 (9 %)	34 (25 %)	18 (13 %)	66 (11 %)	*χ* ^2^ = 41.77, *p* < 0.001
DSM-IV disorders, *n* (%)					
Any disorder	352 (8 %)	39 (29 %)	7 (5 %)	60 (10 %)	*χ* ^2^ = 82.61*, p* < 0.001
Anxiety disorder	132 (3 %)	17 (13 %)	6 (4 %)	24 (4 %)	*χ* ^2^ = 45.06*, p* < 0.001
Depressive disorder	44 (1 %)	9 (7 %)	0 (0 %)	12 (2 %)	*χ* ^2^ = 64.22*, p* < 0.001
Attention-deficit disorder	44 (1 %)	4 (3 %)	1 (1 %)	6 (1 %)	*χ* ^2^ = 4.37*, p* = 0.225
Disruptive disorder	176 (4 %)	21 (16 %)	1 (1 %)	24 (4 %)	*χ* ^2^ = 52.36*, p* < 0.001
SDQ score, mean ± SD					
SDQ total score	8.92 ± 0.10	15.19 ± 0.45	9.24 ± 0.41	11.65 ± 0.20	*F* = 26.09*, p* < 0.001
Emotions symptoms	2.23 ± 0.04	3.35 ± 0.20	2.61 ± 0.17	3.04 ± 0.08	*F* = 41.98*, p* < 0.001
Conduct problem	1.77 ± 0.03	3.75 ± 0.17	1.86 ± 0.13	2.35 ± 0.07	*F* = 82.66*, p* < 0.001
Hyperactivity	3.57 ± 0.04	6.19 ± 0.17	3.50 ± 0.18	4.65 ± 0.09	*F* = 91.77*, p* < 0.001
Peer problem	1.35 ± 0.03	1.91 ± 0.14	1.27 ± 0.11	1.62 ± 0.06	*F* = 12.83*, p* < 0.001
Impact	0.17 ± 0.01	1.04 ± 0.12	0.19 ± 0.05	0.41 ± 0.04	*F* = 59.29*, p* < 0.001

### Social aptitude in youths screened positive for an episode of elated mood

Parent-reported manic symptoms were significantly and negatively associated with the social aptitude score (*b* = −3.63, *p* < 0.001). The association remained significant after controlling for age, gender, any DSM-IV psychiatric disorder and social impact (*b* = −1.84, *p* < 0.001) (Table 5). The association between self-reported mania symptoms and the Social Aptitudes Scale was negative but non-significant (*b* = −0.45, *p* = 0.066; after adjustment *b* = −0.14, *p* = 0.564).

**Table 5:** Relation between the social aptitude score and the presence of elated episodes by parent- and self-report

Outcome: social aptitude score	Parent report	Self-report
Model A	*R* ^2^ = 0.03	*R* ^2^ = 0.01
Predictor		
Elated episodes	*b* = −3.63 *p* < 0.001	*b* = −0.45 *p* = 0.066
Model B	*R* ^2^ = 0.22	*R* ^2^ = 0.09
Predictors		
Elated episodes	*b* = −1.84 *p* < 0.001	*b* = −0.14 *p* = 0.564
Age	*b* = 0.22 *p* < 0.001	*b* = 0.27 *p* < 0.001
Gender (female)	*b* = 1.03 *p* < 0.001	*b* = 1.20 *p* < 0.001
Any DSM-IV disorder	*b* = −4.38 *p* < 0.001	*b* = −5.97 *p* < 0.001
SDQ impact score	*b* = −1.60 *p* < 0.001	*b* = −0.27 *p* < 0.001

*b* Non-standardized regression coefficient, *R* ^*2*^ coefficient of determination

### Relations between social aptitude and each dimension of manic symptoms

**Fig. 1:**
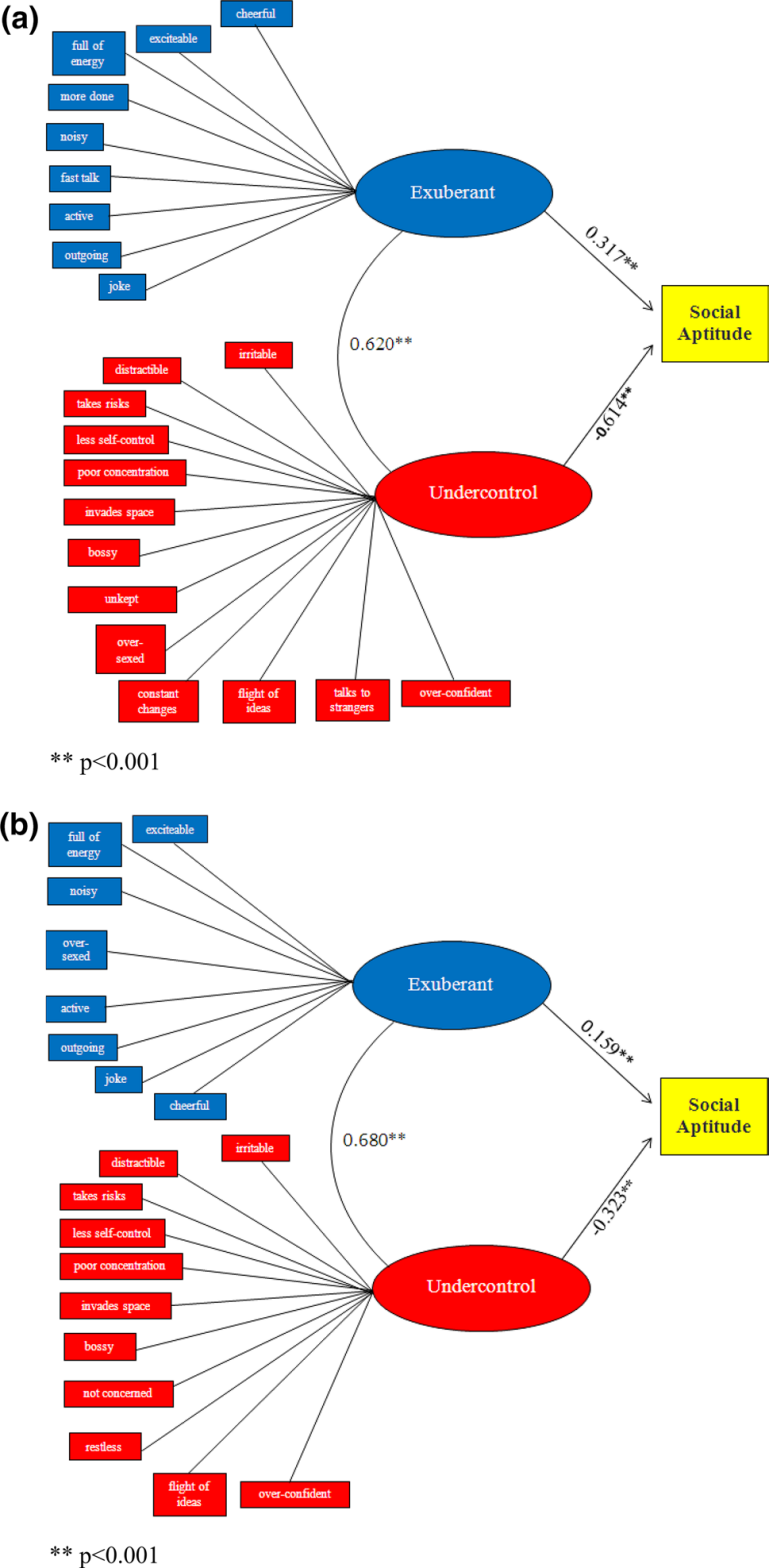
**a** Relations between each dimension of* manic-like* symptoms and social aptitude score (parent report). **p < 0.001. **b** Relations between each dimension of* manic-like* symptoms and social aptitude score (self-report). **p < 0.001

As illustrated in Fig. 1a and b, both dimensions of manic symptoms were significantly associated with the social aptitude score. Social aptitude was positively associated with the exuberant dimension; but negatively with the undercontrol dimension by parent and self-report.

**Fig. 2:**
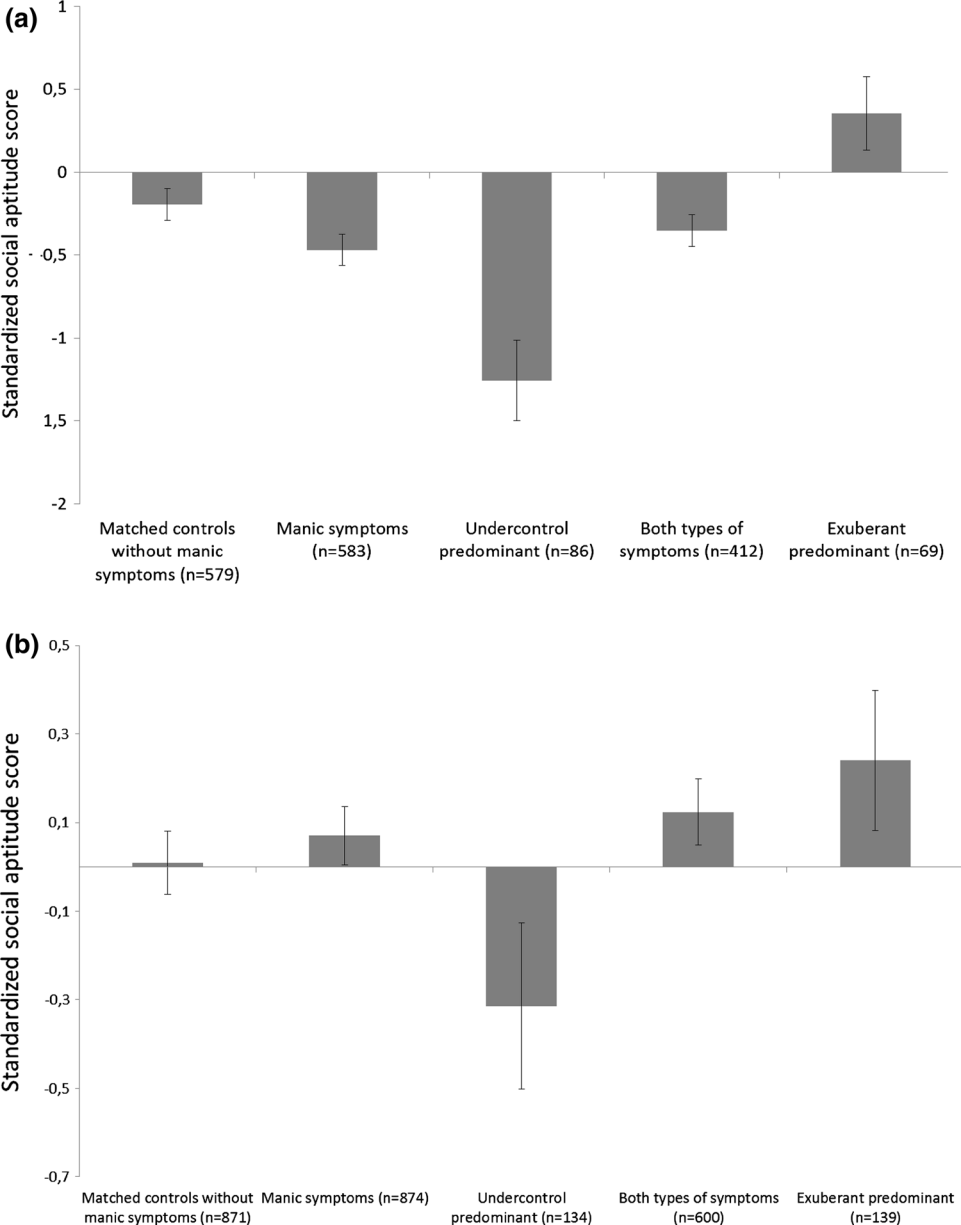
**a** Standardized scores on the Social Aptitude Scale among subjects with parent-reported exuberant, undercontrol and both symptoms of mania, compared to matched controls. **b** Standardized scores on the Social Aptitude Scale among subjects with self-reported exuberant, undercontrol and both symptoms of mania compared to matched controls

As illustrated Fig. 2a, for parent report, the standardized mean score on the Social Aptitudes Scale was different between the “Exuberant” group, the “Undercontrol” group, the “Both types of symptoms” group and the participants without manic symptoms; overall ANOVA: *F* = 82.77, *df* = 3, *p* < 0.001. Post hoc tests revealed that the group with predominantly exuberant symptoms scored significantly higher on the Social Aptitudes Scale (*M* = 0.35, SD = 0.94), than the group with undercontrol symptoms (*M* = −1.26, SD = 1.15, Tukey HSD = 20.13), than the group with both types of symptoms (*M* = −0.35, SD = 0.99, Tukey HSD = 8.82), and the rest of the population (*M* = −0.20, SD = 1.15, Tukey HSD = 3.43). The group with predominantly undercontrol symptoms had a significantly lower score compared to the group without manic symptoms (Tukey HSD = 9.72).

We found similar results for self-reported manic symptoms, as shown in Fig. 2b. For self-report (overall ANOVA: *F* = 10.00, *df* = 3, *p* < 0.001): group with predominant exuberant symptoms scored significantly higher on the Social Aptitudes Scale (*M* = 0.24, SD = 0.95) compared to the group with undercontrol symptoms (*M* = −0.31, SD = 1.09, Tukey HSD = 8.89) and the group without mania symptoms (*M* = 0.01, SD = 1.03, Tukey HSD = 3.61), but was comparable to the group with both types of symptoms (*M* = 26.77, SD = 0.94, Tukey HSD = 1.85). In contrast, the group with predominant undercontrol symptoms scored significantly lower compared to the group without mania symptoms (Tukey HSD = 4.29).

## Discussion

We used parent- and self-reported data from a national survey of 5–16-year-olds to examine the association between social aptitude and manic symptoms. In keeping with our first hypothesis, we found that children and adolescents with parent-reported manic symptoms have a lower social aptitude score compared to the general population. However, youths with self-reported manic symptoms were comparable to the general population with regard to social aptitude. Results were consistent after controlling for age, gender and the presence of any DSM-IV psychiatric disorders.

In accordance with our second hypothesis, social aptitude was positively associated with the exuberant manic dimension, and negatively with the undercontrol one, by parent and self-report. Moreover, children with predominantly exuberant manic symptoms displayed a higher level of social aptitude compared to the matched control group; whereas, as expected, the youths scoring high on the undercontrol dimension of symptoms had a lower score. As youth with manic symptoms present with lower social aptitude overall, it is likely that the negative effects of undercontrol symptoms outweigh the positive effects of the exuberant symptoms.

In our sample, lower social aptitude scores were only found in youth who screened positive for episodes of elated mood based on parent but not self-report. This could be due to statistical reasons. Indeed, the Social Aptitudes Scale is a parent-reported questionnaire; therefore, the generally higher correlation reported within informants as opposed to across-informants could explain our non-significant finding when using self-reported information on mood symptoms. Moreover, previous studies noted that youths with manic symptoms tend to underreport their symptoms compared to parental report [40].

The distinct relationships between each dimension of manic symptoms and social aptitude have several possible explanations.

First, different levels of social aptitude among children with manic symptoms could lead to distinct behavioural manifestations. For example, children with the highest level of social aptitude may be more likely to engage in specific behaviours to share pleasure during an episode of elated mood and may be considered lively, warm, or enthusiastic. In contrast, children who have greater difficulty in understanding social roles of others might be less inclined to modulate their own mood state according to contextual expectations during an episode of elated mood (e.g. by the teacher in a classroom). The distribution of each dimension in clinical and non-clinical populations, as well as the stability over time of each dimension needs to be evaluated to test this hypothesis.

Second, it may be that only certain manic-like symptoms impact the level of social aptitude in youths presenting with an episode of elated mood. For example, Stringaris et al. [29] examined dimensions of manic symptoms among 1755 adolescents from the non-clinical IMAGEN cohort (mean age 14.4 years); youths were screened with a similar method. Only undercontrol, but not exuberant symptoms dimension, was associated with a lower level of functional impairments. In addition, only the undercontrol dimension was specifically associated with poor performance on a response inhibition task. If confirmed, such specific neurocognitive impairments, may partly explain why the undercontrol symptoms dimension was negatively associated with the level of social aptitude in the present study. Indeed, the capacity to inhibit inappropriate responses has been regarded as an essential developmental step to manage positive social relationships in youth [34, 41]. Moreover, a poor ability to inhibit motor responses may be particularly harmful for social interactions in contexts associated with frustration. Therefore, youths with undercontrol symptoms could be more prone to peer rejection and social withdrawal [42, 43]. Further studies should investigate whether such deficits in social interactions extend beyond the period of mood elation, as suggested in adults [1].

By contrast, symptoms associated with the exuberant dimension of manic symptoms may foster social interactions; this would explain the positive association between the exuberant dimension and social aptitude in this study. For example, being more cheerful (item 1 of bipolar section of DAWBA) or joking more than usual (item 18 of bipolar section of DAWBA) may ease social interactions with peers. This finding is consistent with previous research concerning possible positive outcomes associated with (hypo)manic symptoms in clinical and non-clinical populations of children, adolescents, and adults. Indeed, studies exploring the dimensional structure of manic symptoms highlighted a “bright” side of hypomania associated with socially positive and advantageous outcomes [24]. Gamma et al. [44] showed that adults (mean age 40 years) presenting with a “pure” hypomanic episode (without depressive symptoms) earned more money and were more likely to be married compared to the general population. Holtmann et al. [25] investigated a non-clinical sample of 294 adolescents (mean age 17.3 years) using the HCL-32. They distinguished between those who were “active/elated”, “disinhibited/stimulation-seeking”, and “irritable–erratic” and found that the active/elated hypomanic symptoms dimension was negatively associated with peer problems. In a non-clinical sample of 103 adolescents (mean age 17.9 years), higher total HCL-32 scores were related to current early-stage intense romantic love, and to developmental tasks such as exploring and learning psychosocial behaviours [24, 45]. Exuberant dimension of manic symptoms was associated with a wide range of behaviours that fostered positive social interactions with peers. Based on previous research suggesting that exuberance may underlie superior performance in adults, Stringaris et al. [29] found that among youths with an episode of elated mood, the presence of an exuberant dimension of manic symptoms was positively and independently associated with verbal IQ. Besides, a large amount of empirical work stressed the relationship between verbal IQ and social competence development (e.g. having close relationships, being popular with others) during childhood [46]. It could, therefore, be speculated that the higher level of social aptitude observed in youth with exuberant manic symptoms compared to those without manic symptoms is partly mediated by higher cognitive abilities, such as verbal intelligence.

Our findings have a number of potential implications. From an aetiological perspective, our study emphasises the need to expand our understanding of the mechanisms underlying different dimensions of manic symptoms. Clearly the two dimensions are correlated, but as we have shown, there are also “pure” cases of each dimension and investigating them further may prove useful in understanding the heterogeneity in bipolar outcomes more generally. Also, while we offer some hypotheses about the possible mechanisms underlying the difference in social aptitude between exuberance and undercontrol, more work needs to be done to test these and other possible assumptions. From a clinical perspective, practitioners may gain useful insights by considering exuberance and undercontrol manic symptoms as separable dimensions. For example, psychological interventions—such as family-based interventions [47] focus on reducing negative aspects of interaction such as expressed emotion. Our findings indicate that interventions may also focus—for at least some people with manic symptoms—on symptoms of exuberance and their potentially superior social aptitude. In analogy to reinforcing positive behaviour in parenting interventions, positive manifestations related to exuberance could be strengthened and encouraged to generalise, potentially through reward learning. Also, clinicians may want to be mindful about the effects that their treatments, particularly pharmacological, may have on social aptitude. It is plausible to assume that as medication treatment may reduce symptoms of exuberance, this may be perceived as unpleasant by patients and their social environment. Such effects can be discussed between the patient and the clinician to enhance compliance with medication.

This study has number of strengths, such as the use of a large epidemiological sample and the assessment of a broad range of manic symptoms by two reporting sources. This study also has limitations. First, the design of our study does not allow determining the direction of effect between manic symptoms and social aptitude. Future studies should employ prospective data collection and multiple assessment time points. Secondly, our measure of social aptitude is only based on parent report. Despite the fact that the Social Aptitudes Scale is a standardized and validated measure [31], it would be useful to corroborate the findings using information obtained from different informants (e.g. teachers) and other measures of social competence (e.g. acceptance among peers group). Thirdly, in this study, functional impairment and duration criteria of manic symptoms have not been used to define the sample. Therefore, caution is required to extrapolate these findings to youths presenting with a manic episode. As previously noted, the nature of manic symptoms exhibited could substantially differ between inpatients and youths from community-based samples, if only those with specific types of symptoms (e.g. predominantly undercontrol symptoms) seek medical assistance [44]. Fourthly, concerns have been raised regarding the difficulties distinguishing symptoms of elated mood from extraverted temperament in young people. Unfortunately, no data were available about individual temperamental characteristics in this study. As the screening question focused on episodic symptoms, which involved a change in usual functioning, it should exclude youths with persistent high levels of activity which reflects an extraverted temperament. Finally, depressive symptoms prior to the evaluation were not assessed. Considering the impact of depressive symptoms on social functioning over the course of bipolar disorder reported in adults [48], evaluation of past depressive symptoms would be of great interest to examine how it relates to each dimension of manic symptoms and how they may impact social skills.

In conclusion, using a large community-based survey, we found that children with manic symptoms had lower parent-reported social aptitude compared to the general population. This result masked distinct relations between social aptitude and each manic dimension. Children with manic symptoms had a low social aptitude score when parent-reported undercontrol symptoms were predominant; but a high score in case of predominantly exuberant parent-reported symptoms. Our results provide further evidence for the distinction between exuberant and undercontrol manic symptoms and highlight the need to focus on social aptitude in future research.

## Electronic supplementary material

Below is the link to the electronic supplementary material.

Supplementary material 1 (DOCX 13 kb)
