# Supplementary material for: Distinct relationships between social aptitude and dimensions of manic-like symptoms in youth
Source: Eur Child Adolesc Psychiatry. 2015 Dec 9;25:831–42. doi: 10.1007/s00787-015-0800-7 (PMC4967092; doi:10.1007/s00787-015-0800-7)
Supplement: Supplementary file 1 — Supplementary material 1 (DOCX 13 kb) [file 787_2015_800_MOESM1_ESM.docx]

Table S1. Bipolar S Section of DAWBA.

Screening question: “Some young people have episodes of going abnormally high. During these episodes they can be unusually cheerful, full of energy, speeded up, talking fast, doing a lot, joking around, and needing less sleep. These episodes stand out because the young person is different from their normal self.” They were then asked: “Do you [Does Name] ever go abnormally high?”.

| Item | Description |
| --- | --- |
| 1 | More cheerful than usual |
| 2 | Talking faster than normal |
| 3 | More active than normal |
| 4 | Getting things done faster than usual |
| 5 | Noisier than usual |
| 6 | More likely to spend any money that you have as soon as you get it |
| 7 | Able to sleep less than usual without being tired the next day |
| 8 | Restless, unable to keep still |
| 9 | Over-sexed in your talk or behaviour |
| 10 | Constantly changing plans or activities |
| 11 | Full of energy |
| 12 | More likely to start conversations with strangers |
| 13 | More excitable than usual |
| 14 | Less concerned if you get into trouble |
| 15 | More likely to invade other people’s personal space |
| 16 | Over-confident, thinking too highly of yourself |
| 17 | More likely to take serious risks |
| 18 | Joking and laughing more than usual |
| 19 | More outgoing, chatty and sociable than usual |
| 20 | More irritable, with more angry outbursts (which may lead to arguments or fights) |
| 21 | More easily distracted by things going on around you |
| 22 | Less able to stop yourself from doing things you enjoy but know you shouldn’t do |
| 23 | Less able to concentrate |
| 24 | Too bossy with other people |
| 25 | Less concerned about your appearance (clothes, hair etc) |
| 26 | Hard to follow when you are talking because you jump so rapidly from topic to topic |
